# Removal of inferior vena cava filter by open surgery after failure of endovenous retrieval

**DOI:** 10.3389/fcvm.2023.1127886

**Published:** 2023-04-17

**Authors:** Xuan Tian, Jianlong Liu, Jinyong Li, Wei Jia, Peng Jiang, Zhiyuan Cheng, Yunxin Zhang, Xiao Liu, M. I Zhou, Chenyang Tian

**Affiliations:** Department of Vascular Surgery, Beijing Jishuitan Hospital, Beijing, China

**Keywords:** vena cava filter, open surgical filter removal, pulmonary embolism, thrombosis, complication

## Abstract

**Background:**

The permanent placement of inferior vena cava (IVC) filters may lead to numerous complications and their removal is recommended once the risk of pulmonary embolism is reduced. Removal of IVC filters by endovenous means is preferred. But failure of endovenous removal happens when recycling hooks penetrate the vein wall and filters are left in place for too long time. In these scenarios, open surgery may be effective for removal of IVC filters. We aimed to describe the surgical approach, outcomes, and 6-month follow-up of the removal of IVC filter by open surgery, after the failure of removal *via* the endovenous method.

**Methods:**

A total of 1,285 patients with retrievable IVC filters were admitted from July 2019 to June 2021, including 1,176 (91.5%) endovenous filter removals, and 24 (1.9%) open surgical IVC filter removals after the failure by endovenous method, of whom 21 (1.6%) were followed-up and eligible for analysis of the study. Patient characteristics, filter type, filter removal rate, IVC patency rate, and complications were retrospectively analyzed.

**Results:**

Twenty-one patients were left with IVC filters for 26 (10, 37) months, of which 17 (81.0%) patients had non-conical filters and 4 (19.0%) had conical filters; all 21 filters were successfully removed, with a 100% removal rate, no deaths, no serious complications, and no symptomatic pulmonary embolism. At the 3rd month follow-up after surgery and 3rd month follow-up after discontinuation of anticoagulation therapy, only 1 case (4.8%) had IVC occlusion, but without any occurrence of new lower limb deep venous thrombosis and silent pulmonary embolism.

**Conclusion:**

Open surgery can be used for the removal of IVC filters after failure of removal by endovenous method or when accompanied by complications without symptoms of pulmonary embolism. Open surgical approach can be used as an adjunctive clinical intervention for the removal of such filters.

## Background

Lower limb deep venous thrombosis (DVT) is a common condition, requiring prompt treatment, and the dislodged thrombus may lead to a pulmonary embolism that is a life-threatening condition (1–3). It has an incidence of approximately 1–2/1,000 people (4–6). Currently, inferior vena cava (IVC) filters are used clinically to prevent fatal pulmonary embolism during the perioperative period (7–9). Indications for vena cava filter placement in the Chinese guidelines (10) are floating thrombus in the iliac, femoral or inferior vena cava, acute DVT that needs thrombectomies such as catheter-directed thrombolysis (CDT), pharmacomechanical thrombectomy (PMT), or surgical thrombectomy, and abdominal, pelvic, or lower limb surgery with high-risk factors for acute DVT and pulmonary embolism (PE). Permanent placement of IVC filters, however, may lead to numerous complications (11–13), and therefore, it is recommended (8, 9, 14, 15) that retrievable vena cava filters can be placed perioperatively and removed when the risk of pulmonary embolism is reduced.

Most IVC filters are removed by endovenous method, but hooks in recycling conical filters penetrate the vein wall, resulting in failure of endovenous retrieval of the filter; non-conical filters cause rapid endothelial proliferation and increased difficulty of endovenous retrieval after retention for more than 14 days (16–18), however forced retrieval of the filter may damage the IVC. The filter required been retained for a prolonged period in cases of pulmonary embolism risk, IVC embolism, or floating thrombus in the proximal iliac femoral vein (19). Failure to undergo medical intervention on time or other unknown factors may also increase the filter retention time and increase the risk of filter removal.

IVC filters after failure of removal by endovenous means may be removed by open surgery. This retrospective study aimed to describe the surgical approach, outcome, and experience with the removal of IVC filter by open surgery after failure of removal using the endovenous method.

## Methods

### General information

**Figure 1:**
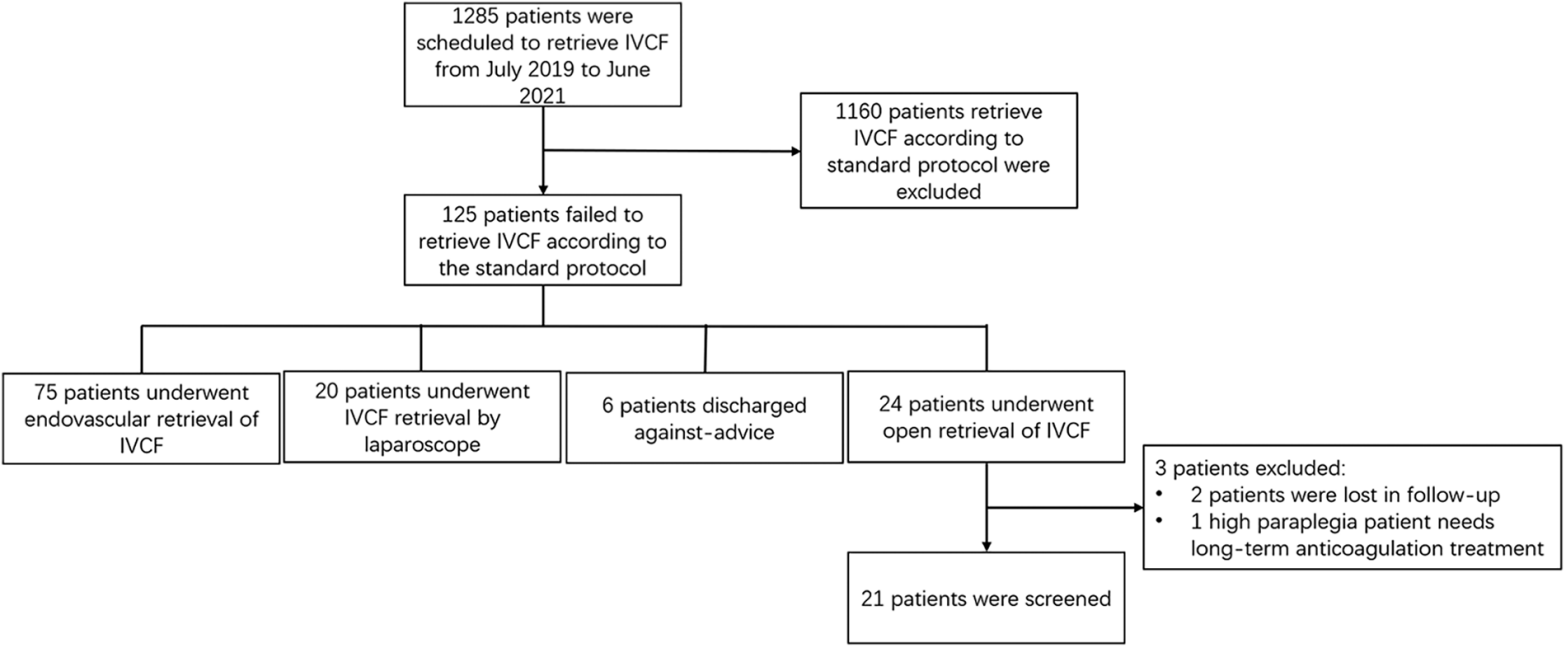
Flow chart of patients who choose open surgery to remove inferior vena cava filter.

We retrospectively analyzed 24 (1.9%) filter retrieval cases by open surgery from July 2019 to June 2021, 21 cases (1.6%) were completed successfully,3 patients were excluded, 2 patients were lost in follow-up after surgery,1 case with high paraplegia from thoracic spine trauma, discontinuation of anticoagulation therapy due to high risk of thrombosis recurrence, all above patients received filter implantation in other medical centers (Table 1 and Figure 1).

**Table 1:** Characteristics of filters and patients’ general condition.

Characteristics of filters and patients’ general condition	Results
**Overall filter removal**	
Total number (cases)	1,285
Denali	803 (62.5%)
Option	26 (2.0%)
Temper filter	41 (3.2%)
Celect	49 (3.8%)
Tulip	7 (0.5%)
Cordis	323 (25.1%)
LifeTech	36 (2.8%)
Transfer from other hospitals after the failure of removal (cases)	125 (9.7%)
Conical filter (cases)	63 (50.4%)
Removal by intracavitary method	45 (71.4%)
Removal by laparoscope (20)	11 (17.5%)
Removal by open surgery	4 (6.3%)
Against-advice discharge^a^	3 (4.8%)
Non-conical filter (cases)	62 (49.6%)
Removal by intracavitary method^b^ (19)	39 (62.9%)
**Open removal of the filter**	
Age (years)	43.4 ± 13.0
Gender (male)	10/21 (47.6%)
BMI	24.1 ± 1.8
**Previous history (cases)**	
Coronary heart disease	1/21 (4.8%)
Diabetes	1/21 (4.8%)
**Reason for placement of filter (cases)**	
Pulmonary embolism	4/21 (19.0%)
Deep venous thrombosis	17/21 (81.0%)
Fracture surgery	12/17 (70.6%)
Pregnancy	3/17 (17.6%)
Other	2/17 (11.8%)
**Reason for not removing filter on time (cases)**	
Risk of pulmonary embolism	5 (23.8%)
Longer treatment for thrombus	8 (38.2%)
Thrombus in the filter	4 (19.0%)
Patients’ factors^c^	4 (19.0%)
Duration of filter retention (months)	26 (10, 37)
**Type of retained filter (cases)**	
Cordis	10/21 (47.6%)
LifeTech	7/21 (33.3%)
Celect	2/21 (9.5%)
Tulip	2/21 (9.5%)
**Reason for open filter removal (cases)**	
Fracture	2 (9.5%)
Recyclable hook penetrating the venous wall	2 (9.5%)
Abdominal pain	6 (28.6%)
Refusal for permanent anticoagulation therapy	5 (23.8%)
Psychological factors	6 (28.6%)

^a^ Refusal of further treatment after failure of intracavitary retrieval.

^b^ Including removal by placing a new filter.

^c^ Including failure to seek medical intervention on time, fear of surgery, and other psychological reasons.

Three patients discontinued anticoagulation therapy after 3 months, 8 cases took rivaroxaban 20 mg QD for anticoagulation, and 10 cases took rivaroxaban 10 mg QD for anticoagulation. The study was approved by the Ethics committee of Beijing Jishuitan Hospital (No.JST202201–21).

Removal of filters by open surgery is not a routine procedure and is mostly used in patients with a broken or displaced filter, or with abdominal pain and after the failure of endovenous removal. Selection criteria were as follows: patients aged <70 years, with no contraindications to anticoagulants, and no serious cardiopulmonary disease that would prevent the procedure; IVC is patent at least in unilateral iliac veins, with no thrombus or retaining a small amount of old thrombus in the lower limb veins and IVC, above situations can be considered as low risk of pulmonary embolism after removal of the filter; failure to retrieve the conical filter by endovenous method because of severe tilt or CT scan showing the penetration of filter hook into the IVC wall; patients did not accept permanent placement of a filter or had a strong desire for filter retrieval or accept of filter retrieval by open surgery.

### Treatment

Preoperative examinations and symptom observation: Ultrasonography of lower limb veins and IVC was performed to observe the patency of IVC, patency of bilateral iliac veins or at least unilateral iliac veins, absence of thrombus in both lower limb veins or presence of old thrombus, and the risk of thrombus dislodgement. Enhanced CT imaging of the abdomen was performed to observe the patency of IVC, the condition of the filter (broken or not), the penetration of retrievable hook into the vascular wall (21), and the presence of symptoms of pulmonary embolism. The signs of pulmonary embolism were also observed.

Intraoperative manipulation: The surgical operation was performed by a physician with a senior title in vascular surgery. Under general anesthesia, a longitudinal incision was made on the right rectus abdominis; the hepatocolic ligament was severed *via* the lateral right paracolic sulcus; the right hemi-colon was lifted to the retroperitoneum; the proximal and distal ends of the IVC were freed; the lumbar vein was separated and ligated (special attention was paid to the lumbar vein in the posterior wall of the IVC) to avoid damage to the duodenum, iliac vein, and right ureter; surrounding tissues were protected to minimize damage when separating the retrievable hooks or foot supports of the penetrated filters.

**Figure 2:**
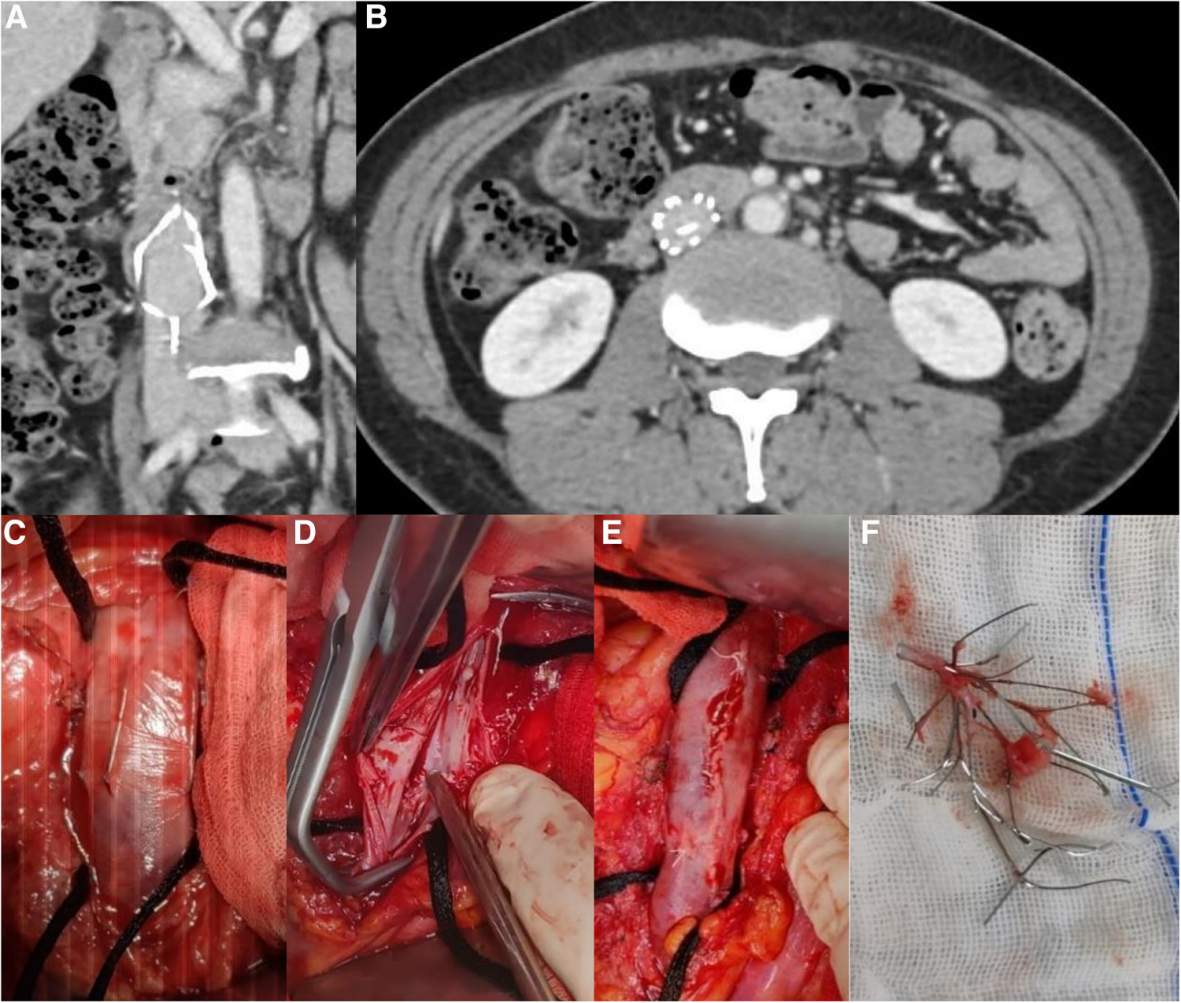
(**A**) Retention of non-conical filter for 8 years (**B**) Filter fracture (**C**) Filter penetrating the IVC (**D**) Post-repair image of IVC endothelium after filter removal (**E**) Suture of the IVC (**F**) Removed filter after disassembly.

**Figure 3:**
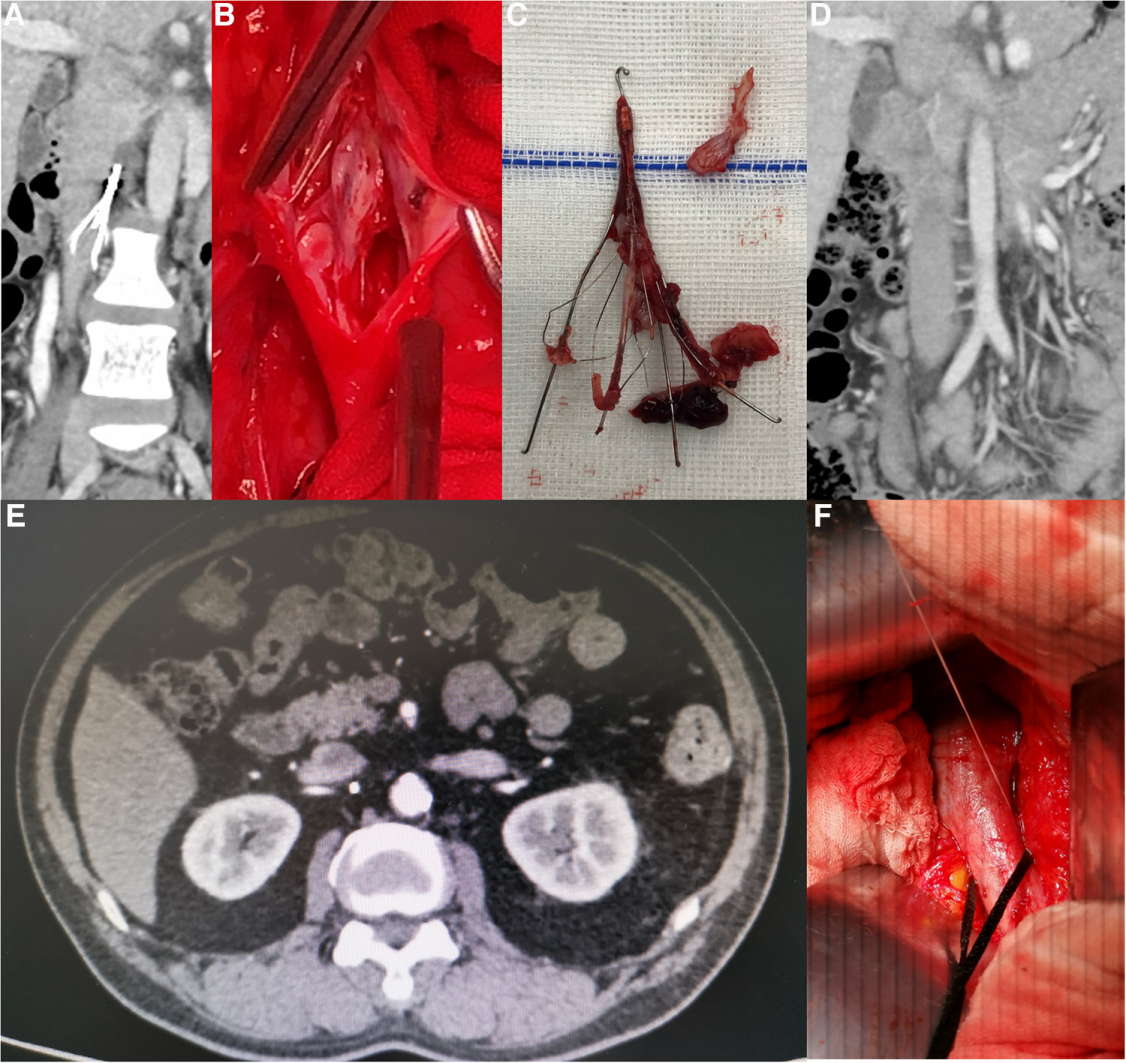
(**A**) Retention of Tulip filter for 16 months. (**B**) Tulip filter with obvious endothelial hyperplasia and failed endovenous removal. (**C**) Complete removal of Tulip filter with severe endothelial hyperplasia. (**D**) Follow-up of Tulip filter removal at 6 months after surgery with patent IVC. (**E**) CT image of the Celect filter showing the retrievable hook penetrating the IVC. (**F**) Intraoperative retrievable hook penetrating the venous wall with a preplaced purse-string suture.

The following two methods were chosen depending on the types of filters: a) Non-conical and Tulip filters: The IVC was blocked after systemic heparinization; the filters were separated by longitudinal dissection of the anterior wall of the IVC; the endothelium of the IVC was repaired and IVC was then sutured (Figures 2, 3A-D). b) For conical filters other than Tulip filters: The penetrated venous wall by the retrievable filter hooks was preplaced with purse-string suture; The filter can be easily removed or with the aid of a 10F arterial sheath tube after incision of the inferior vena cava along the retrieval filter hook, followed by a purse-string ligation to stop bleeding (22) (Figures 3E-F).

Postoperative treatment: Low molecular weight heparin at the dose of 100 IU/Kg Q12h was administered for anticoagulation. Patients were discharged with the prescription for oral administration of rivaroxaban at a dose of 20 mg QD for anticoagulation (23), with plasma D-dimer monitoring at 1-month intervals.

At 3rd month postoperative follow-up (September 2019 to September 2021), venous ultrasound of both lower limbs was performed to observe the thrombotic changes in the limbs and enhanced CT of the abdomen was performed to observe patency of the IVC. The symptoms of pulmonary embolism were monitored. After discontinuing anticoagulation therapy for 3 months (December 2019 to December 2021), an ultrasound examination of both lower limbs and IVC was performed to observe the patency of IVC and thrombotic changes in the limbs.

### Statistical analysis

SPSS version 21.0 software was used for statistical analysis. The measurement data were expressed as mean ± standard deviation, the count data were described as percentages, and the non-normally distributed measurement data were expressed as median (M) (P25, P75); the Wilcoxon rank-sum test was used for comparison between the groups, and the difference was considered statistically significant when *P*-value was <0.05.

## Results

### IVC filters

A total of 1,285 patients were admitted for retrieval of retrievable filters, of which 926 (72.1%) patients had conical filters, including 871 (94.1%) cases of endovascular retrieval, and 359 (27.9%) had non-conical filters, including 305 (85.0%) cases of endovascular retrieval. The overall retrieval rate was 91.5% (1,176/1,285) by endovascular method (Table 2).

**Table 2:** Information of retrieval vena cava filters.

Type	Total cases	Nonretrieval cases	Reasons for not retrieving the filters		
			Thrombosis	tilt/penetration	lost in follow-up/voluntarily discharged against medical advice
Denali	803 (62.5%)	28 (3.5%)	21 (75.0%)	0	7 (25.0%)
Option	26 (2.0%)	1 (3.8%)	1 (100%)	0	0
Temperfilter	41 (3.2%)	0	0	0	0
Celect	49 (3.8%)	5 (10.2%)	2 (40%)	2 (40%)	1 (20%)
Tulip	7 (0.5%)	1 (14.3%)	0	1 (100%)	0
Cordis	323 (25.1%)	73 (22.6%)	29 (39.7%)	1 (1.4%)	43 (58.9%)
Lifetech	36 (2.8%)	1 (2.8%)	1 (100%)	0	0
Total	1285	109 (8.5%)	54 (49.5%)	4 (3.7%)	51 (46.8%)

**Figure 4:**
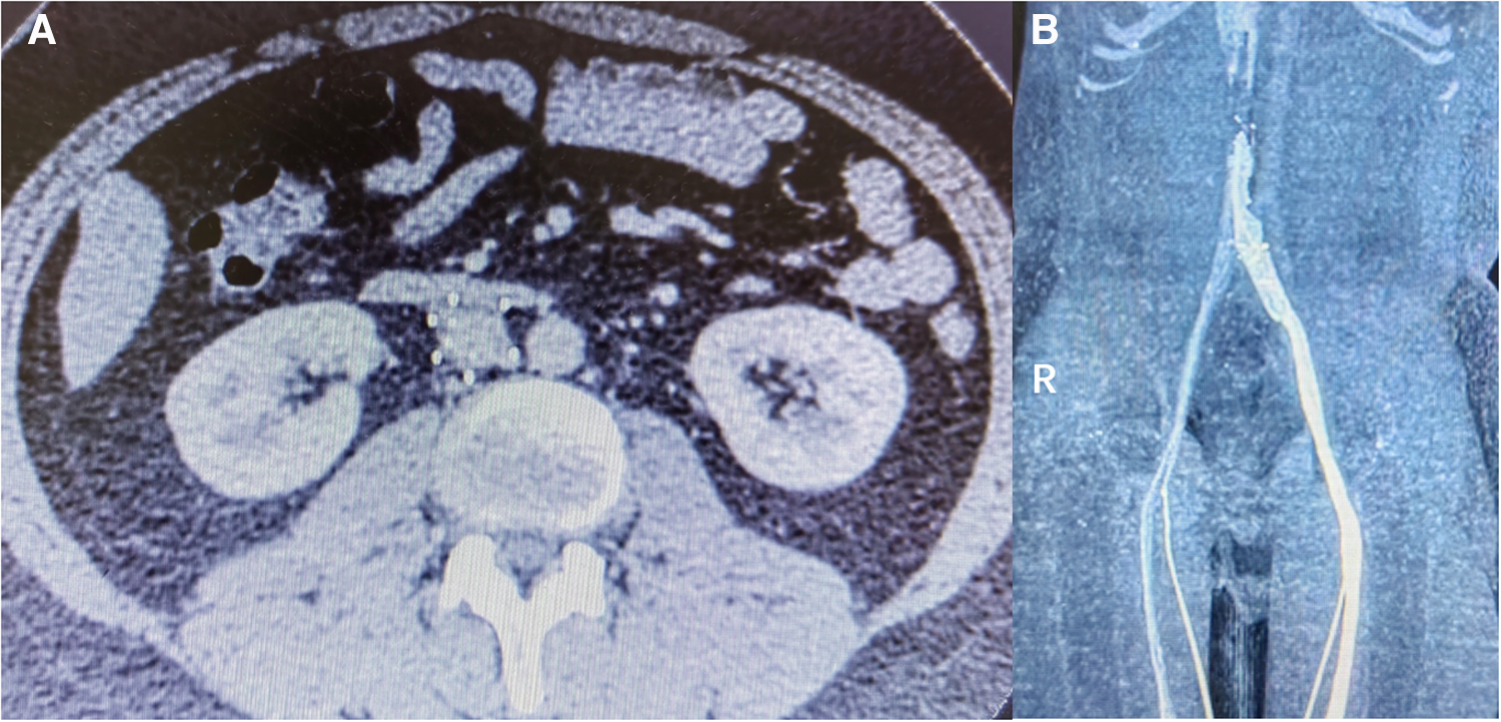
(**A**) Retention of non-conical filter for 6 years, fractured. (**B**) IVC occlusion in the follow-up at 3 months after surgery.

125 patients (9.7%) were transferred to our hospital after the failure of endovascular retrieval of IVC filters, 24 (1.9%) patients underwent filter retrieval by open surgery, and 21 (1.6%) cases completed the surgery and follow-up. Of the 21 IVC filters left for 26 (10, 37) months (Table 3), 17 (81.0%) were non-conical, 2 (9.5%) of them displayed fractures on CT images (Figures 2C, 4B), did not penetrate the venous wall, and there was no hematoma around the IVC (Figure 2A); of the remaining 16 patients with non-conical filters, 10 had unsuccessful removal by endovenous attempts in other hospitals within two weeks of admission to our hospital and refused further removal by endovenous method, 6 were removed with complications by attempts in our hospital, 3 were removed with abdominal pain, and 3 were removed by double wire lassoing techniques. Four (19.0%) conical filters (Tulip, 2; Celect, 2) did not show a fracture, and removal attempts were made using the loop technique, modified loop technique, and assisted balloon dilation technique, but these techniques were unsuccessful for Celect filters and CT image showed the penetration of retrievable hooks into the IVC (Figures 2A,E). retrievable hooks were captured in Tulip filters, which were not successfully removed due to severe abdominal pain, and removal by endovenous means was abandoned.

**Table 3:** Intraoperative and postoperative conditions and follow-up.

Intraoperative and postoperative conditions and follow-up	Results	Z/t	*P*
Operation duration (min)	223.3 ± 23.1		
Filter removal (cases)	21/21 (100%)		
Length of stay (days)	11.0 ± 1.9		
ICU duration (h)	26.6 ± 13.1		
Ventilator use duration(h)			
Exhaust time (h)	49 (44, 52)		
Start of drinking water (h)	49 (44, 52)		
Start of eating (h)	60 (56, 70)		
**Change in hemoglobin (g/L)**			
Before surgery	130 (124, 137)^a^	4.204	0.001
1 day after surgery	113 (110, 121)^b^	1.046	0.308
7 days after surgery	117 (107, 120)	1.457	0.153
Blood transfusion	5/21 (23.8%)		
ALT change (IU/L)	15 (12, 18)		
Before surgery	18 (13, 29)		
After surgery			
AST change (IU/L)		1.681	0.101
Before surgery	16 (14, 17)	1.567	0.125
After surgery	18 (15, 24)		
CR change (μmoI/L)	60 (55, 64)		
Before surgery	55 (48, 60)		
After surgery			
**Change in D-Dimer (mg/L)**			
Before surgery	0.30 (0.22, 0.42)^a^	4.023	0.001
1 day after surgery	1.88 (1.52, 2.68)^b^	2.030	0.056
7 days after surgery	3.42 (2.30, 5.86)		
**Change in Fibrinogen (mg/dl)**			
Before surgery	262.8 (235.0, 293.0)^a^	4.336	0.000
1 day after surgery	365.0 (289.2, 453.6)^b^	2.954	0.008
7 days after surgery	458.5 (367.0, 617.8)		
**Postoperative complications (Clavien-Dindo)**			
Grade I	16/21 (76.2%)		
Grade II	5/21 (23.8%)		
Grade III-V	0/21 (0%)		
Wound infection	0/21 (0%)		
Catheter-associated infection	0/21 (0%)		
Amylase	0/21 (0%)		
Urinary retention	0/21 (0%)		
Gross hematuria	0/21 (0%)		
Myocardial infarction	0/21 (0%)		
Heart failure	0/21 (0%)		
Respiratory failure	0/21 (0%)		
Pneumonia	0/21 (0%)		
Pulmonary atelectasis	0/21 (0%)		
Pleural effusion	0/21 (0%)		
Perioperative deaths (cases)	0/21 (0%)		
Follow-up time (months)	7.7 ± 3.4		
Recurrent lower limb deep venous thrombosis (cases)	0/21 (0%)		
New IVC thrombosis (cases)	1/21 (4.8%)		
Pulmonary embolism symptoms (cases)	0/21 (0%)		

^a^ Comparison of preoperative and 1 day postoperative.

^b^ Comparison of changes at 1 day and 7 day postoperative.

### Surgical procedure

Open surgery was performed to remove the IVC filters within 1–2 weeks of the failure of removal by the endovenous method. Twenty-one filters were successfully removed with a 100% removal rate (Table 3), no perioperative deaths, and no symptomatic pulmonary embolism. The wound healed well after surgery without serious complications (Table 3). Hemoglobin content comparisons before surgery and 1 day and 7 days after surgery were as follows: 130 (124, 137), 113 (110, 121) and 117 (107, 120) g/l; for the comparison before surgery and 1 day and after surgery, *Z* = 4.204 and *P* = 0.0001; for the comparison at 1 day and 7 days after surgery, *Z* = 1.046 and *P* = 0.308. Comparison of biochemical indicators before surgery and 3 days after surgery were as follows: glutamic-pyruvic transaminase, 15 (12, 18) and 18 (13, 29) and µmol/l, *Z* = 1.457, *P* = 0.153; glutamic oxalocetic transaminase, 16 (14, 17) and 18 (15, 24) µmol/L, *Z* = 1.681, and *P* = 0.101; creatinine, 60 (55, 64) and 55 (48, 60) μmoI/L, *Z* = 1.567, and *P* = 0.125. This indicated that there was a small amount of bleeding during the filter removal by open surgery technique and after carefully applying hemostasis, no further blood was lost and liver or renal function was not affected after surgery.

### Postoperative follow-up

Ultrasonography of the lower limbs and CT imaging of the IVC were performed 3 months after continuous anticoagulation therapy. Twenty-one cases showed no new acute lower limb deep venous thrombosis, 20 cases had patent IVC (Figure 3D) and 1 case (4.8%) had IVC occlusion (Figure 4B).

Anticoagulation therapy was discontinued in 20 cases, except for the case with IVC occlusion. On the reexamination 3 months after discontinuation of anticoagulation treatment, the IVC was patent, there was no new acute lower limb deep venous thrombosis, and no symptomatic pulmonary embolism occurred. Oral administration of rivaroxaban at a dose of 20 mg QD was continued for anticoagulation in the case with IVC occlusion. No complications due to anticoagulation therapy occurred in all patients.

## Discussion

Sequelae of permanent filter placement may lead to complications and cause a serious physical and psychological impact on patients and their families (16, 24) who have strong desire to have the filters to be removed, in China. Complications of long-term filter retention are vena cava perforation, movement of filter support adjacent to or penetrating into surrounding organs, and filter fracture with symptoms due to fracture-associated risk (25–28).

### Results and implications

Due to the complications associated with long-term retention of filters, our center attaches great importance to the retrieval of filters. The overall filter retrieval rate in our study was 91.5%, and the reasons for failure to retrieve filters were 54 (49.5%) The reasons for failure to retrieve the filter were as follows: inferior vena cava thrombosis or intra-filter thrombosis in 54 (49.5%) cases, missed visits or automatic discharge in 51 (46.8%) cases, and severe tilting of the filter inducing the hook against the wall in 4 (3.7%) cases, mainly due to thrombosis and missed visits.

One of the main risks of filter removal by open surgery is hemorrhage, mainly occurring in lumbar and iliac veins, due to a large number of tributaries from IVC. The lumbar veins at the anterior and lateral walls of the IVC are easy to find, but those at the posterior wall of the IVC are difficult to separate and can be easily damaged. The injury to the lumbar veins may be reduced by ligating the tissue near the posterior wall of the IVC in stages during surgery. Blocking the IVC immediately distal to the filter is the main method of reducing hemorrhage from the iliac vein tributaries, and preoperative CT imaging assessment is important.

The IVC is located posterior to the peritoneum and another risk of open surgery is that it interferes with the intestinal function. The surgery was performed *via* the lateral right hemi-colon to the retroperitoneum without disrupting the mesenteric and intraperitoneal vessels, thus reducing the impact on the blood supply to the gastrointestinal tract and without causing internal hernia.

Other complications may occur in open surgery to retrieve the filter: ①inferior vena cava thrombosis and pulmonary embolism intraoperative and postoperative. In order to reduce vena cava injury, strict heparinization (100 IU/Kg) before blocking the vena cava is needed. Strict anticoagulation therapy was administered for 3 months following surgery, anticoagulation could be discontinued after a repeat CT scan hinting no stenosis or occlusion in inferior vena cava. In our study, there is no symptomatic pulmonary embolism and no new lower limb deep venous thrombosis. In the follow-up at 3 months after surgery, the IVC was patent in 20 cases and occluded in 1 case. The cause of the occlusion was considered to be related to endothelial hyperplasia and filter fracture after long-term retention of the filter. In the follow-up at 3 months after discontinuation of anticoagulation therapy, the IVC was still patent in 20 cases. Open surgical removal of the IVC filter was associated with a low risk of pulmonary embolism, discontinuation of anticoagulation after repair of the IVC endothelium. ② intraoperative injury to the inferior vena cava, common injury include adhesions between the barb penetrated the vein and the surrounding tissue, and injury due to separation of the filter from the vein wall. The filter can be cut and removed to reduce the damage to the vein intraoperative, and the injure of vena cava can be repaired with sutures. If the defect of vein is too large to be repaired, an autologous vein or an artificial patch, or even reconstruction with an artificial vena cava replacement is preferred. In this study, there was no serious injury to the inferior vena cava occurred in the patients and the injure of vena cava could be repaired by direct suture.

Rigorous preoperative screening and adequate thrombosis management are required. Before surgery, adequate screening should be performed to select patients who have thrombus formation, who have a reduced risk of pulmonary embolism, who have the good inflow and outflow tracts on CT scans, and who are at low risk of developing IVC thrombosis or new acute deep venous thrombosis in the presence of good blood flow. During the surgery, it is important to ligate the lumbar vein on the side of the IVC adjacent to the lumbar spine to significantly reduce hemorrhage. After surgery, anticoagulation therapy may be discontinued after 3–6 months with a patent IVC and no new acute deep venous thrombosis.

### Comparison of related filter retrieval techniques

The “Loop snare technique”(29) is often used to correct the tilt of a tapered filter and to retrieve the filter when the retrieval hook is attached to the endothelium. However, the “Loop snare technique” is not successful in retrieving the filter when the retrieval hook is adhered to the inner membrane. In above cases, “the Hangman technique” can be used to retrieve the filter by cutting the adherent endothelium tissues, but it will also be limited by the availability of a localized slit to pass through. “The Loop snare technique” and “the Hangman technique” were used in all four cases of conical filters enrolled in this study and were unsuccessful in retrieving the filters, so the filters were retrieved by open surgery.

The laparoscopic technique of filter removal is more suitable for conical filter adhered to the vein wall with less damage than open surgery, but is limited in two ways: (i) whether the retrieval hook has penetrated the vein wall; if the retrieval hook has not penetrated the vein wall, it is difficult to locate the retrieval hook intraoperatively; and (ii) where the retrieval hook has penetrated the vein wall; when the retrieval hook penetrates the posterior wall of the inferior vena cava, it is more difficult to separate the posterior wall of the inferior vena cava using the laparoscopic technique. The non-conical filter and the vein wall are more heavily lined and the separation process is likely to damage the vein wall, and the non-conical filter has not yet been removed using laparoscopic techniques. In the four cases included in this study, the conical filter was removed by open surgery because the penetration site was in the posterior or lateral posterior wall.

### Comparison of relevant studies

Tunner et al. (30) reported 190 IVC filter removals with severe complications in multiple clinical centers, of which 90 were removed using endovenous method and 100 were removed by open surgery. Of the 100 open surgery removals, 45 were performed *via* thoracotomy approach and 55 were performed *via* laparotomy approach. Open surgery led to a higher incidence of venous thromboembolism(VTE) complications, whereas no recurrence of IVC and lower limb deep venous thrombosis, and no symptomatic pulmonary embolism were found in the present study. Tunner et al.'s article reported a 5% mortality rate for open surgery, whereas no deaths occurred in the present study, which may have been attributed to the different characteristics of the cases analyzed in the study. Patients in the present study were mainly cases after the failure of removal of the filter by endovenous means, with 2 cases who had fracture complications, whereas cases reported by Tunner et al. were all with complications such as fractures and filter displacements. The cases were recruited in our study were malfunction of the filter and presented with comorbidities including respiratory, gastrointestinal, renal function, infection or thromboembolism. Our study focused on patients with filter rupture, abdominal pain, refusal of permanent anticoagulation or psychological factors, and some patients did not develop filter complications, which may be related to psychological factors in the Chinese population.

Rana (31) reported 6 cases of open surgical filter retrieval, the inferior vena cava was clamped in 2 cases, the inferior vena cava was not clamped in 3 cases, and the broken pedicle was removed successfully in 1 case. The unclamped inferior vena cava surgical approach was mainly applied to tapered filters with the retrieval hook penetrated through the vein wall, and the reserved suture around the retrieval hook could sealed the inferior vena cava after the filter was removed. In our study, the inferior vena cava was clamped intraoperatively in all patients, including 17 cases of non-conical filters and one case of Tulip filter removal using a similar method, but the inferior vena cava was still clamped and no inferior vena cava thrombosis or injury occurred after surgery.

### Limitations

CT pulmonary angiogram (CTPA) was not performed before or after open surgery to remove the IVC filter, and no imaging was done to assess the presence of asymptomatic pulmonary embolism before or after surgery. Also, this was a single-center retrospective study with small sample size, and no control group was established.

### Summary

In conclusion, open surgery can be used to remove IVC filters in cases after failure of endovenous removal attempts or with complications and as an adjunct intervention for successfully removing filters while avoiding the risk of serious injury to the IVC and relieving the physical and psychological distress in patients without symptomatic pulmonary embolism.

## Data Availability

The raw data supporting the conclusions of this article will be made available by the authors, without undue reservation.
